# Quantum transport on honeycomb networks

**DOI:** 10.1038/s41598-022-10537-w

**Published:** 2022-04-27

**Authors:** Geyson Maquiné Batalha, Antonio Volta, Walter T. Strunz, Mircea Galiceanu

**Affiliations:** 1 Departamento de Física, Universidade Federal do Amazonas, Manaus, 69077-000 Brazil; 2 ARPAE-SIMC, viale Silvani 6, 40122 Bologna, Italy; 3 Institut für Theoretische Physik, Technische Universität Dresden, 01062 Dresden, Germany

**Keywords:** Quantum physics, Quantum optics

## Abstract

We study the transport properties on honeycomb networks motivated by graphene structures by using the continuous-time quantum walk (CTQW) model. For various relevant topologies we consider the average return probability and its long-time average as measures for the transport efficiency. These quantities are fully determined by the eigenvalues and the eigenvectors of the connectivity matrix of the network. For all networks derived from graphene structures we notice a nontrivial interplay between good spreading and localization effects. Flat graphene with similar number of hexagons along both directions shows a decrease in transport efficiency compared to more one-dimensional structures. This loss can be overcome by increasing the number of layers, thus creating a graphite network, but it gets less efficient when rolling up the sheets so that a nanotube structure is considered. We found peculiar results for honeycomb networks constructed from *square* graphene, i.e. the same number of hexagons along both directions of the graphene sheet. For these kind of networks we encounter significant differences between networks with an even or odd number of hexagons along one of the axes.

## Introduction

Transport properties of materials and structures are an active field of research in physics, chemistry, biology, and computer science. Many such phenomena of complex dynamics in the classical realm can be modeled by a classical Random Walk (RW)^1–5^. Problems such as the configurational properties of polymers^6^, kinetic chemical reactions^1,7^, diffusion^8,9^, or epidemic spreading of diseases^10,11^ are explained through RW models. For a growing number of phenomena, however, quantum effects can no longer be neglected. Thus, in the last years, efforts have been tremendous to consider quantum mechanical variants of the RW, of which Quantum Walks (QWs) on networks are a prime example. This framework can be related to a number of real-world problems, such as coherent and incoherent quantum dynamics of excitations in complex systems^12–16^, dynamics on quantum graphs^17–19^, quantum search algorithms, quantum information or quantum computation^20–27^. By exploiting the interference of quantum waves, combined with measurement, quantum search algorithms show a polynomial or even an exponential increase in the speedup^28^, when compared to their corresponding classical counterparts. This impressive result has additionally started an avalanche of experimental work related to this problem^23,29–47^.

Similar to the classical case, quantum transport^28,48^ can be studied by using two distinct models: the discrete-time quantum walk (DTQW)^49^ and the continuous-time quantum walk (CTQW)^50^. It was shown that these two models are not independent^51^. Moreover, there are alternatives to these two basic models, such as the quantum stochastic walk^52–54^ and a Green’s function approach for quantum graphs^55,56^. In the CTQW model, which is our chosen model, the dynamics of the quantum walk is solved by identifying the Hamiltonian with the transfer matrix of the network, which is proportional to the discrete Laplacian. As in the classical model, in the CTQW model the transfer matrix of any undirected network is associated to the connectivity matrix^2^ of the underlying graph. Then, all quantum transport properties can be obtained directly from its eigenvalues and eigenvectors. We evaluate the efficiency of transport through the probability to return to the starting node after a given time, and also consider the corresponding long-time average return probability.

Continuous-time quantum walks have been studied on a great number of network structures^57–68^. Here, we use honeycomb networks, motivated by graphene structures. Since the successful isolation of a single sheet of graphene^69^, many of its unusual properties^70–76^ were encountered and exploited in various applications^71–75,77–80^. In this report we consider quantum transport (CTQWs) on graphene and its most relevant carbon structures, motivated by the fact that nowdays, graphene and their derivates can be obtained in laboratory and they can serve as a non trivial architecture to compare theoretical and experimental findings.

Other theoretical works were devoted to quantum walks on honeycomb or graphene-based structures. In Ref.^81^ the problem of localization on honeycomb structures was studied by using the DTQW model, while the same model was implemented to graphene related structures to determine the accumulated arrival probability^82^. The problem of quantum search on graphene lattice by making use of a CTQW model was addressed in Refs.^27,83,84^. A discrete-time staggered quantum walk model was used to compute the mean-square displacement on hexagonal lattices and to solve the spatial search problem^85^. The article^86^ focuses on excitation source-to-sink transport for percolated coined quantum walks on nanotubes. It was shown in Ref.^87^ that for the line, square and honeycomb lattice topologies there is a unitary equivalence of coined and scattering discrete time quantum walk models. The scattering construction of discrete time quantum walks on honeycomb lattices was studied in detail^88^, obtaining ten independent models. By contrast to all these works, we monitor in detail the evolution of quantum efficiency on honeycomb networks in the CTQW framework, monitoring its dependence on the topology of the structures. We find that the transport is strongly influenced by the underlying topology of the network.

It is known that in our model the quantum transport on networks with many branches, such as Cayley trees or stars^57,58^, shows strong localization effects. The situation is different if one considers the CTQWs on linear chains, for which we encounter a very good spreading over the network^57^. Honeycomb networks like graphene and its related structures display a mixture of linear and starlike segments. For the considered networks the number of branches is very small, namely three for all nodes of fullerenes, two or three for graphene sheets or nanotubes, and ranging between two and five for graphite. Thus, the number of branches is not as low as in rings, for which we have only two, or as high as in scale-free networks^59^, for which one can consider highly connected nodes. All our networks are composed of connected rings of size six, i.e. hexagons. Additionally, we also have some pentagons for fullerenes and plenty of squares for graphite networks. Note that this variety of topologies cannot be captured in terms of the topological directional functions^88^. We will monitor how the quantum transport efficiency changes by stacking one graphene sheet on top of each other, similar to the procedure developed in Ref.^60^, or by rolling-up cylinders of graphene forming nanotubes.

The paper is organized as follows: In “Methods”, we present the construction of our honeycomb networks based on graphene structures and we provide a brief description of the continuous-time classical and quantum walk models implemented in the paper. In “Results”, we show results for fullerenes, graphene, graphite, and nanotubes, focussing on the eigenvalue spectrum of the Laplacian (connectivity) matrix, the classical and quantum return probabilities and the long time average probability. The “Discussion” will end this paper.

## Methods

### Honeycomb networks

**Figure 1:**
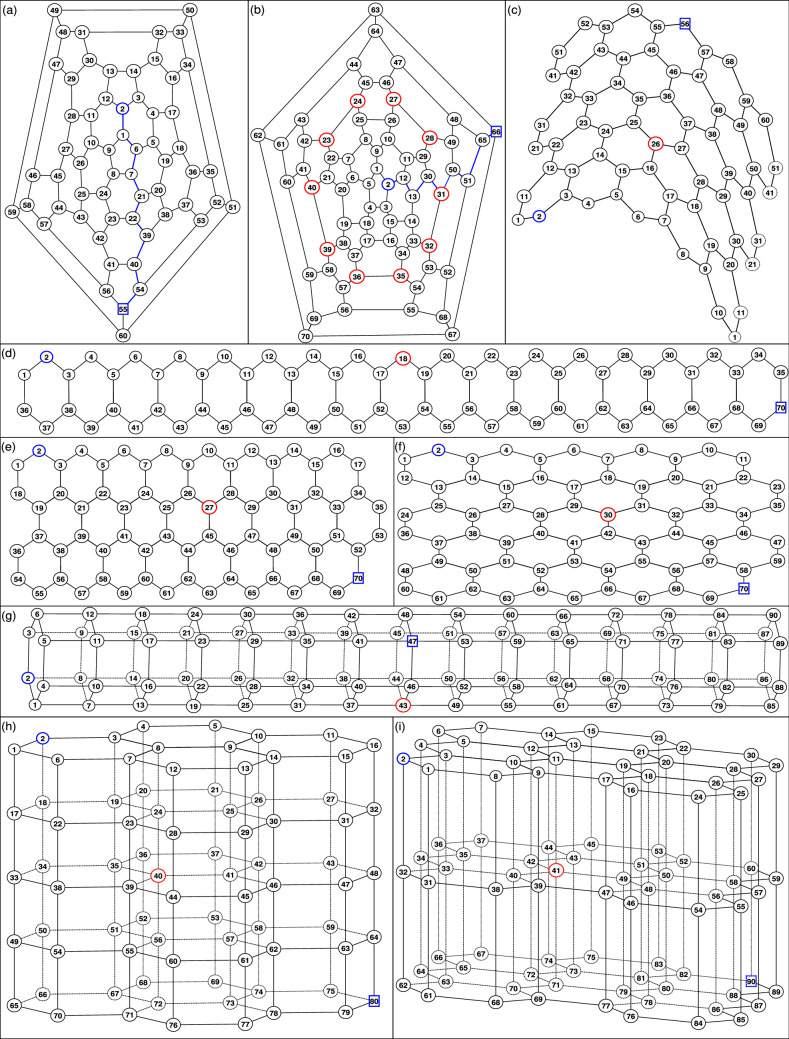
Examples of honeycomb networks: Fullerenes: (**a**) $C_{60}$ and (**b**) $C_{70}$, which are represented by using the Schlegel representation^114^. Nanotube: obtained by rolling up a graphene-type network: a network with $(L_x,L_y)= (10,6)$ (**c**) is displayed. Graphene-type flat networks with $(H_x,H_y)=(17,1)$ (**d**), (8, 3) (**e**) and (5, 5) (**f**). Graphite-type networks built as multilayer graphene network with $(H_x,H_y,L)=(1,1,15)$ (**g**), (2, 2, 5) (**h**), and (3, 3, 3) (**i**).

Honeycomb structures are ubiquitous as carbon is one of the most present chemical elements on earth. Structures based on graphene sheets gained more importance in the last decades with the discovery of its multifaceted properties^69^, related to the dimensionality of its different possible topologies. From this large variety of structures we choose to analyse the most symbolic ones: fullerenes, graphene, graphite, and nanotubes. Fullerenes, known also as buckyballs resemble a hollow sphere^89^. From the physical point of view, they are considered finite, zero-dimensional objects with discrete energy states. Since its discovery in 1985 many theoretical as well as experimental studies were devoted to $C_{60}$. They are strongly related to carbon nanotubes and both of them received attention in the nano-scale research area. Here, we consider the most known alotropes of carbon: $C_{60}$ and $C_{70}$, although others were synthesized^90,91^ or encountered in the universe^92,93^. $C_{60}$ and $C_{70}$ are formed by nodes with 3 links each and their faces are hexagons or pentagons, thus, one could easily relate them to graphene. The ratio between faces type can be found from Euler’s polyhedron formula^90^: $N+F-E=2$, where *N* is the number of nodes, *F* is the number of faces and *E* gives the number of links (edges). From this formula any fullerene has exactly 12 pentagons and $N/2-10$ hexagons. In Fig. 1a,b we display networks derived from $C_{60}$ and $C_{70}$, respectively. All the nodes were numbered in order to facilitate the understanding of the results.

Graphene is a two-dimensional allotrope of carbon and it is viewed as a single layer of atoms arranged as a honeycomb network made up of hexagons. Although it is widely considered as the cornerstone of all known allotropes of carbon, a single graphene sheet was only recently isolated^69^. Since then, many experimental and theoretical studies continue to uncover its peculiar properties^94–105^, to cite only a few. In this article we use honeycomb networks mimicking a sheet of graphene defined in terms of the number of hexagons along two directions, $H_x$ and $H_y$. In Fig. 1d–f we display three small graphene-type networks with $(H_x,H_y)=(17,1), (8,3),$ and (5, 5), respectively. The numbering is as follows: first we give a number to nodes from the first line of the *y*-axis by moving along the *x*-axis, then we move to the second line along the *y*-axis and so on until the last node of the network. We choose this method of numbering for reasons which will become clear in the “Results” section. From the construction procedure it is clear that we will not have dangling nodes; all nodes belong to at least one hexagon. The total number of nodes $N_{G}$ and links $L_{G}$ for a graphene honeycomb network with $H_x$ hexagons along the *x*-axis and $H_y$ hexagons along the *y*-axis are given by

1 $$\begin{aligned} N_{G}=2(H_x+H_y+H_xH_y), L_{G}=N_G+H_xH_y-1. \end{aligned}$$

These equations are derived by observing that the number of links along the *x*-axis equals $2H_x+1$ (peripheral lines) and $2H_x+2$ (inner lines).

Layers of graphene can be piled on top of each other creating graphite. This is the three-dimensional allotrope of carbon and it is one of the most stable form of carbon under standard conditions. It possesses properties of both metals and non-metals, having high thermal and electrical conductivity^106^ and the layers can easily move past each other due to weak forces between adjacent layers. By using Raman spectroscopy it was proven that changes in its electronic structure allows to identify the graphene layers^107^. Two adjacent layers are usually arranged in such a way that the atoms in one of the two sublattices of the honeycomb structure of one layer are situated above one half of the atoms in the neighboring layer^95^. In this article we construct our multilayer honeycomb network as a sequence of identical copies of the initial layer. We also consider that all the nodes from a layer are connected to a single node from the neighboring layers, thus, each node from a layer is situated directly above a node from their adjacent layers. Such networks appear also in graphite intercalated compounds^108,109^ or they can be obtained by layer-by-layer assembling techniques^110^. In Fig. 1g–i we display three graphite networks with $(H_x,H_y,L)=(1,1,15), (2,2,5),$ and (3, 3, 3), respectively. Here, we keep the parameters used for graphene, namely $H_x$ and $H_y$ give the number of hexagons along the *x*- and *y*-axis, respectively, and we denote by *L* the number of identical layers. First, we put the numbers on nodes from the first layer and then we move to the second layer and so on. The numbers of the nodes of each layer are given according to the procedure described above for a single sheet graphene network. The total number of nodes and links of a graphite-type honeycomb network are therefore given by

2 $$\begin{aligned} N_{S}=L N_G,\, L_{S}=L L_G+ N_G (L-1), \end{aligned}$$

where $N_G$ and $L_G$ are given by Eq. (1).

Carbon nanotubes can be obtained by rolling a single layer of graphene along a certain rotational axis and reconnecting the open bonds. The nanotubes are composed only of hexagons and thus, from the physical point of view they can be considered one-dimensional structures^95^. Carbon nanotubes have a large surface area and high thermal and electrical conductivity^111^. In the literature, two classes of nanotubes are distinguished: single-wall carbon nanotubes (single layer of graphene), and multiwall carbon nanotubes (several layers). Here, we consider the first class, for which there are three ways of wrapping a honeycomb network to a cylindrical honeycomb tube, so-called armchair, chiral, and zigzag type^112^. Here, we construct our nanotube from the graphene by cutting the last bonds from each line and reconnecting it to the first node of the line, corresponding to the zigzag type^112^. Due to this prescription, which considers lines instead of hexagons, we choose to change our parameters to $L_x$ and $L_y$, which give the number of nodes of each line along both directions. However, it is important to stress that the new parameters are related to $H_x$ and $H_y$ through the following simple relations:

3 $$\begin{aligned} H_x=\frac{L_x}{2},\, H_y=L_y-1, \,N_N=L_x L_y,\, L_N=\frac{L_x(3L_y-1)}{2}, \end{aligned}$$

where $N_N$ and $L_N$ denote the total number of nodes and links of the nanotube-honeycomb network. In Fig. 1c we display a nanotube-network with $L_x=10$ and $L_y=6$.

### Theoretical model

In this article we study quantum transport on honeycomb networks by making use of the CTQW model. For a better understanding of transport properties we study also the corresponding classical model, the CTRW. Here, we consider a network to be a set of *N* nodes, which are coupled to other nodes by links. In this formalism we associate to each node a state $|j\rangle$, $j=1,\ldots ,N$ which physically can be seen as an excitation localized at node *j*.

The dynamics on any network for both a CTRW and a CTQW depends only on the connections between the nodes. All transport properties are determined from the complete set of eigenvalues and eigenvectors of the so-called *connectivity matrix* $\mathbf{A}$. This matrix is an $N \times N$ real and symmetric matrix, having its nondiagonal elements $A_{jk}$ equal to $-1$ if there is a link between nodes *j* and *k*, and 0 otherwise. Its diagonal elements equal the number of links emerging from node *j*, namely $A_{jj}=k_j$. It is clear^57^ that we encounter only positive real eigenvalues $\lambda _n$ except for a single vanishing eigenvalue, $\lambda _{1}=0$.

For a classical CTRW we start from a Markov process master equation^1^ for the transition probability $p_{j,k}(t)$ to walk from node *k* to node *j* in time *t* ,

4 $$\begin{aligned} \frac{d}{dt} p_{j,k}(t)=\sum _{l} T_{jl} p_{l,k}(t), \end{aligned}$$

where $T_{jl}$ is the transition rate between nodes *l* and *j* and the initial conditions are $p_{j,k}(0)=\delta _{jk}$. For CTRW on a network, we choose the transfer matrix to be proportional to the connectivity matrix, $\mathbf{T}= -\mathbf{A}$, which amounts to consider an equal transition rate for all bonds, which we choose to be 1.

The transition probability of a CTRW can be written as a function of the eigenvalues $\lambda _n$ and the eigenstates $|\Phi _n\rangle$ (with $n=1,\dots ,N$) of the connectivity matrix:

5 $$\begin{aligned} p_{j,k}(t)=\sum _{n=1}^{N} \exp (-\lambda _n t) \langle j|\Phi _n\rangle \langle \Phi _n|k\rangle . \end{aligned}$$

For *quantum* transport we assume a set of orthonormal and complete states $|j\rangle$^50,57^. The dynamics is determined from the quantum mechanical Hamiltonian, such that the Schrödinger equation for the transition amplitudes $\alpha _{j,k}(t)=\langle j|\exp (-i \mathbf{H} t)|k\rangle$ reads

6 $$\begin{aligned} \frac{d}{dt} \alpha _{j,k}(t)=-i \sum _{l} H_{jl} \alpha _{l,k}(t). \end{aligned}$$

The quantum transition probability from node *k* to node *j* at time *t* is given by $\pi _{j,k}(t)=\left| \alpha _{j,k}(t) \right| ^2=\left| \langle j|\exp (-i \mathbf{H} t)|k\rangle \right| ^2$. In CTQW on networks the Hamiltonian is identified with the connectivity matrix^50^: $\mathbf{H}=\mathbf{A}$. Accordingly, the quantum transition probability can again be written as a function of the eigenvalues $\lambda _n$ and the eigenstates $|\Phi _n\rangle$ of $\mathbf{H}$:

7 $$\begin{aligned} \pi _{j,k}(t)=\left| \sum _{n=1}^{N} \exp (-i \lambda _n t) \langle j|\Phi _n\rangle \langle \Phi _n|k\rangle \right| ^2. \end{aligned}$$

In this article we focus on the average return probability and its long-time average as measures for the transport efficiency. The probability to remain or return to the initial node *k* is averaged over all nodes. For CTRWs we set

8 $$\begin{aligned} \overline{P}(t)=\frac{1}{N} \sum _{k=1}^{N} p_{k,k}(t), \end{aligned}$$

and for CTQWs we consider

9 $$\begin{aligned} \overline{\pi }(t)=\frac{1}{N} \sum _{k=1}^{N} \pi _{k,k}(t), \end{aligned}$$

accordingly. For CTRWs by inserting Eq. (5) into Eq. (8), we obtain the average return probability, which turns out to depend on the eigenvalues of the connectivity matrix only:

10 $$\begin{aligned} \overline{P}(t)=\frac{1}{N} \sum _{k=1}^{N} \exp (-\lambda _k t). \end{aligned}$$

In the quantum model we insert Eq. (7) into Eq. (9) and after some manipulations we find that the average return probability depends on both eigenvalues and eigenstates. However, if one makes use of the Cauchy-Schwarz inequality, a lower bound for $\overline{\pi }(t)$, which doesn’t depend on the eigenstates, is found:

11 $$\begin{aligned} \begin{aligned} \overline{\pi }(t)&=\frac{1}{N} \sum _{k=1}^{N} \left| \alpha _{k,k}(t) \right| ^2 \ge \left| \frac{1}{N} \sum _{k=1}^{N} \alpha _{k,k}(t) \right| ^2 \\&=\left| \overline{\alpha }(t)\right| ^2=\left| \frac{1}{N} \sum _{k} \exp (-i \lambda _k t)\right| ^2. \end{aligned} \end{aligned}$$

In this article we consider the average return probabilities, $\overline{P}(t)$, given by Eq. (10), and $\overline{\pi }(t)$, given by Eqs. (9) and bounded by (11). For a better understanding of the results we stress that a fast decay of $\overline{P}(t)$ or $\overline{\pi }(t)$ means a fast spreading of the walker, while a slow decay corresponds to a slow propagation. In the long-time limit we encounter two distinct situations. For CTRWs we find the equipartition value 1/*N*. In the quantum CTQW case, however, $\overline{\pi }(t)$ does not converge to a constant value, but it shows an oscillatory pattern around the long time asymptotic average value^57^:

12 $$\begin{aligned} \chi \equiv \lim _{t \rightarrow \infty } \frac{1}{t} \int _{0}^{t} dt' \left| \overline{\alpha } (t')\right| ^2 \le \lim _{t \rightarrow \infty } \frac{1}{t} \int _{0}^{t} dt' \overline{\pi }(t') \equiv \overline{\chi }. \end{aligned}$$

It is known^59,61^ that the long time average transition probability depends only on the eigenvalue density $\rho (\lambda )$^59,61,62^:

13 $$\begin{aligned} \chi = \sum _{\lambda } \rho ^2 (\lambda ) \ge \rho ^2 (\lambda ^*) + \frac{1}{N} \left[ 1-\rho (\lambda ^*)\right] \equiv \chi ^*, \end{aligned}$$

where $\lambda ^*$ is the most degenerate eigenvalue.

Quantum transport is considered to have maximum efficiency for $\chi =0$ and it is completely inefficient when $\chi =1$. It is also known that the simpler $\chi ^*$ provides a very good approximation of $\chi$ in two extreme situations: for star and linear chain configurations^61^. For a star with $N-1$ neighbors we have $\lambda ^*=1$ as the highest degenerate eigenvalue, with degeneracy $N-2$, which gives $\chi =\chi ^*=[2+(N-2)^2]/N^2$. For very large stars we obtain $\chi _{N \rightarrow \infty }^{s}=1$, which reflects an inefficient transport. In the case of a linear chain, all the eigenvalues are nondegenerate, given an eigenvalue density $\rho (\lambda )=1/N$, for every $\lambda$. Inserting these values into Eq. (13), one finds $\chi =\chi ^*=1/N$. For very long chains we have an efficient transport, $\chi _{\infty }^{l}=0$. It should be mentioned that in other situations $\chi ^*$ does not provide a good approximation for $\chi$, as was shown for other networks^60,63^.

We focus on quantities that depend on the eigenvalues $\lambda _n$ of the connectivity matrix, which will be determined numerically or in a semi-analytical manner. The exact values of the whole eigenvalue spectrum depend on the degree distribution and the arrangement of the links between nodes. However, the upper bound of the eigenvalues of any network can be estimated analytically by using, for instance, Eq. (1.9) of Ref.^113^:

14 $$\begin{aligned} \lambda _{max} \le \max \{ 2+ \sqrt{[T(m,n)-2][T(m,l)-2]} \}, \end{aligned}$$

where (*m*, *n*) and (*m*, *l*) are two distinct links of node *m* and $T(m,n)=\frac{deg(m)}{deg(n)} \eta (m) + \frac{deg(n)}{deg(m)} \eta (n)$ with *deg*(*m*) and *deg*(*n*) being the degree of *m* and *n*, respectively, while $\eta (m)$ is the average degree of the adjacent nodes of *m*.

## Results

We now show our results of continuous-time quantum walks on honeycomb networks. First we focus on the fullerene-types, chosing $C_{60}$ and $C_{70}$ as examples. Our second choice is the graphene sheet structure, for which we investigate how the number of hexagons along each direction influences the quantum transport efficiency. The third considered network is the graphite type, for which we monitor how the number of graphene layers affects quantum transport, especially for *square* graphene sheets. Finally, we study carbon nanotube-types of networks obtained from rolling up a graphene sheet, exploring how the width and length of the sheet influences the quantum transport.

### Fullerene-type honeycomb networks

**Figure 2:**
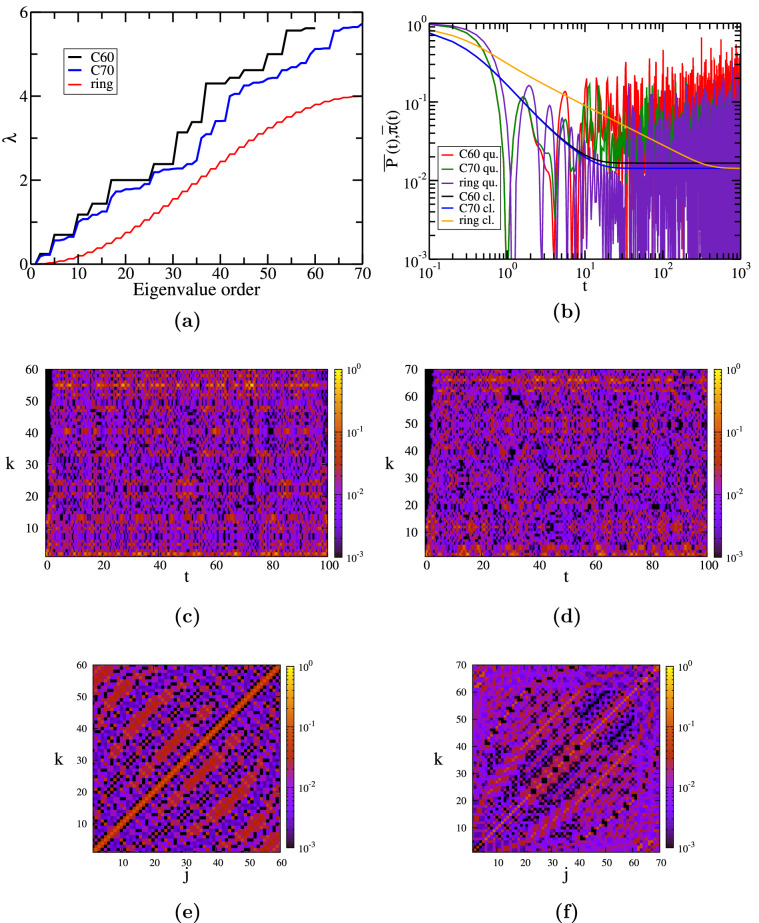
Results for networks of fullerene-type $C_{60}$ and $C_{70}$: (**a**) eigenvalue spectrum, (**b**) classical and quantum return probability. Further, we show the transition probability $\pi _{k,2}(t)$ for $C_{60}$ in (**c**) and $C_{70}$ in (**d**). Finally, we display $\pi _{k,j}(t=10)$ for $C_{60}$ (**e**) and $C_{70}$ (**f**).

In Fig. 2a we plot in progressive order the eigenvalues for the $C_{60}, C_{70}$-networks, and a discrete ring, i.e. a circle, with $N=70$. We notice enormous differences between the spectra of these three structures, which drastically influence quantum transport. The eigenvalue spectrum of $C_{60}$ is highly degenerate due to its symmetry, with $\lambda =2$ being the most degenerate eigenvalue (9-fold). It also has four 5-fold degenerate eigenvalues, three 4-fold degenerate eigenvalues and six 3-fold degenerate eigenvalues. On the other hand, $C_{70}$ and the ring have only non-degenerate or doubly-degenerate eigenvalues. More precisely, the eigenvalue spectrum of $C_{70}$ is formed by 28 doubly-degenerate eigenvalues and 14 non-degenerate eigenvalues. The ring has all the eigenvalues doubly degenerate, with two exceptions for an even number of nodes, namely the vanishing eigenvalue, $\lambda _1=0$, and the largest eigenvalue, $\lambda _{70} \approx 4$. For an odd number of nodes the ring has only one non-degenerate eigenvalue: $\lambda _1=0$, the rest being doubly degenerate. These eigenvalues for a discrete ring can be determined analytically^57^ from the equation $\lambda =2-2 cos \theta$, where $\theta =2 n \pi /N$ with *n* being an integer and $n \in (0,N]$.

In Fig. 2b we display the classical average probability of returning to the starting node $\overline{P}(t)$, as a function of time as given by Eq. (10), and its quantum mechanical equivalent $\overline{\pi }(t)$, Eq. (11). Here, we present the results for $C_{60}$, $C_{70}$, and the ring with $N=70$. The asymptotic behavior of the classical average probability at very long times, $\overline{P}(t)=1/N$, is perfectly recovered for the three structures. This long-time behavior is reached faster by both $C_{60}$ and $C_{70}$ compared to the ring, due to nodes with higher degree. In the intermediate time domain, i.e. times $t>1$, $\overline{P}(t)$ decays as power-law with exponent $-1.0$ for both $C_{60}$ and $C_{70}$, differently from the ring, for which we encounter a power-law behavior with exponent $-0.51$. This value is in good agreement with the power-law exponent found for a very long linear chain^57^, namely 1/2. It is important to stress that networks whose density of states follows a power-law, $\rho (\lambda ) \sim \lambda ^{\mu }$, also show a power law behavior for the classical average probability at not too short times^57^: $\overline{P}(t) \sim t^{-(1+\mu )}$. Here, $2(1+\mu ) \equiv d_s$ is known as the spectral dimension^115^. In the quantum case the envelope of the lower bound for CTQW scales as $env[\left| \overline{\alpha }(t)\right| ^2] \sim t^{-2(1+\mu )}$ for the same kind of density of states^57^. The same scaling was obtained for the decay of temporal correlations in quantum systems with Cantor spectra^116^, where the quantum average probability $\overline{\pi }$ was considered. The quantum average probability $\overline{\pi }$ shows a strong oscillatory behavior for all three structures. For the ring, similar to linear chains, we clearly notice two distinct behaviors: for an intermediate time domain we have a power-law decay with exponent $-1.0$ and for $t \gtrsim 30$ we obtain large fluctuations around the long-time average $\chi _{ring} \approx 0.028$. For fullerenes the long-time behavior is reached faster, $t \gtrsim 10$, due to the fact that we are dealing with more compact structures. We also observe that the fluctuations, fairly large for the ring, are significantly smaller in amplitude. The long-time average $\chi$ is greater for $C_{60}$, namely $\chi _{C_{60}} \approx 0.078$, and a little bit lower for $C_{70}$: $\chi _{C_{70}} \approx 0.025$. Thus, $C_{70}$ shows the highest efficiency for the quantum transport, but it is less than the efficiency of a line with the same amount of nodes^57^, $\chi _{line}=1/N \approx 0.014$. For all considered networks the simple approximation involving $\chi ^*$ does not give good estimates: the relative difference $(\chi -\chi ^*)/\chi$ equals 0.47 (ring), 0.53 ($C_{60}$), and 0.42 ($C_{70}$).

In Fig. 2c ($C_{60}$) and d ($C_{70}$) we focus on the time evolution of the quantum transition probabilities from node 2, $\pi _{k,2}(t)$. Here, we consider times larger than $t \gtrsim 10$ because for earlier times the walk didn’t have enough time to spread throughout the network. For networks of $C_{60}$-type we encounter probabilities greater than 0.2 only for transition to nodes 2 and 55, see Fig. 1 for numbering. Remarkably, the highest probability was found to be $\pi _{55,2}(73.1)=0.69$. For a $C_{60}$-network we found that the highest probabilities to return are $\pi _{2,2}(33.3)=0.34$, $\pi _{2,2}(67.4)=0.37$, and $\pi _{2,2}(81.7)=0.36$, which can be considered as the partial revival times^57^. It is important to stress that node 55 is the most distant node from node 2, i.e. its diametrically opposite node^82^, with the shortest path containing 9 links, depicted by a blue line in Fig. 1. Similar behavior was encountered for all pairs of diametrically opposite nodes, for instance (1, 60) or (6, 59), etc. . This occurrence is reminiscent of the interference on a circle^57^. For circles with an even number of nodes one finds two maximum probabilities: to the starting point and to its opposite node, while for odd-numbered circles we encounter only the first situation. This is due to the symmetry of the network, more exact to the number of steps to go in both directions. In the case of networks of $C_{70}$-type the situation is different. We encounter probabilities higher than 0.2 only for transitions to nodes 2 and 66, but the highest probabilities are lower than in the $C_{60}$ case: $\pi _{2,2}(15.2)=0.32$ and $\pi _{66,2}(39.9)=0.31$. Transition probabilities higher than 0.1 we encounter only for all nodes belonging to the inner and outer pentagons and to nodes 11, 12, and 13. Our results show that node 2 forms a pair with node 66 and we display by a blue line the shortest path between them. For $C_{70}$, a node from the inner pentagon, namely nodes $1-5$, is paired with the nearest node from the outer pentagon. Remarkably, inner nodes also form these kind of pairs, but they are not diametrically opposite, like in the case of $C_{60}$. For example we have the following pairs: (6, 61), (7, 43), (22, 42), to name a few. The most peculiar situation is encountered for nodes surrounded only by hexagonal faces, depicted by red in Fig. 1. For these nodes the highest probabilities range between 0.1 and 0.2 and they correspond to transitions to many different nodes, such as to all red-colored nodes and to their nearest neighbours. The differences between $C_{60}$ and $C_{70}$ can be explained through a symmetry breaking process. The highly symmetric structure of $C_{60}$, which belong to the icosahedral symmetry group $\mathbf{H}_\mathbf{3}$, is transformed to the dihedral symmetry group $\mathbf{H}_\mathbf{2}$ by adding one more decagonal term, creating the $C_{70}$ structure^117^.

In Fig. 2e,f we show all possible transition probabilities, $\pi _{k,j}$, for $C_{60}$ (e) and $C_{70}$ (f) as contour plots at time $t=10$. Similar patterns are observed for other time values. For both fullerene-type networks we find mainly high probabilities to be at the starting node, more evident for $C_{60}$. This aspect validates our findings for the average probability $\overline{\pi }$ and its long-time average probability, $\chi$, namely that the efficiency of transport is better for $C_{70}$. For $C_{60}$ we found that the highest probabilities range between 0.1 and 0.2, corresponding to transition probabilities $\pi _{k,k}$. High probabilities were found also for transitions to diametrically opposite nodes, as discussed in Fig. 2c. For this particular choice of *t* we found that for $C_{70}$ the highest probabilities correspond to transitions between nodes from inner pentagons to nodes from outer pentagons. Other high probabilities were found for transitions between two nearest neighbors of red-colored nodes, for instance $\pi _{21,41}$.

### Graphene

**Figure 3:**
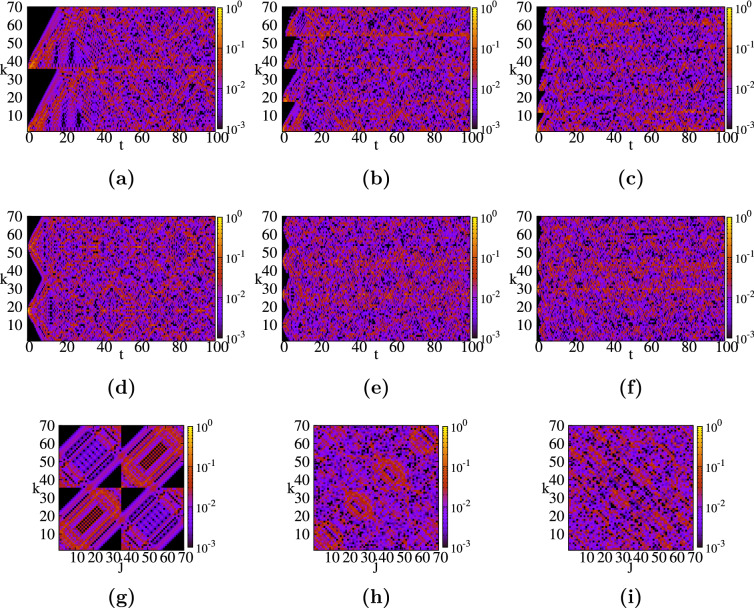
Spacetime structures for small rectangular graphene-type networks ($N=70$) corresponding to three different shapes: (**a**,**d**,**g**) long, quasi-onedimensional network $(H_x=17,H_y=1)$; (**b**,**e**,**h**) medium recangular network $(H_x=8,H_y=3)$; (**c**,**f**,**i**) square network $(H_x=5,H_y=5)$. For each network we consider the return probability $\pi _{k,2}(t)$ (top row), and $\pi _{k,center}(t)$ (middle row) as functions of time, and $\pi _{k,j}(t=10)$ for a fixed time as a function of start and end point of the walker (bottom row).

In Fig. 3 we show the contour plots of the quantum average return probability $\pi$, Eq. (7), of a CTQW that starts at node *k* and ends at node *j* after time *t*. We display the results for the three graphene-type networks shown in Fig. 1, where we number all the nodes to facilitate a better understanding of the results. All of them have $N=70$ nodes, but they differ in the number of hexagons along the *x*-axis, $H_x$, and *y*-axis, $H_y$. For a more comprehensive way to visualize the results we view the coupled hexagons as two lines along *x*-direction, connected to each other through $H_x+1$ links. First we enumerate the nodes from the first line, from left to right, before going to the second line, see Fig. 1. The first choice, whose results are shown in the left column (Fig. 1a,d,g), corresponds to a long network formed by 17 hexagons along the *x*-axis, thus we have set the parameters to $(H_x, H_y)=(17, 1)$. The middle column, Fig. 3b,e,h, correspond to a graphene-type network with $(H_x, H_y)=(8, 3)$. Finally, the third graphene-type network is a *square* graphene sheet with the same amount of hexagons on both directions, $(H_x, H_y)=(5, 5)$, shown in the right column: Fig. 3c,f,i. For the sake of comparison we show the same quantities for all these small graphene-type networks and we choose the same threshold $\pi$-value, namely $10^{-3}$ for the color code. It is worth mentioning that we determine the transition probabilities for times between $0 \le t \le 100$, with a stepsize of 0.1.

In the first row of Fig. 3, i.e. panels (a), (b), and (c), we display the time evolution of the quantum transition probability, for the CTQW to start at the peripheral node 2, depicted by a blue circle in Fig. 1, and to end at node *k* after time *t*, $\pi _{k,2}(t)$. Immediately apparent is the ”ballistic”, linear propagation along the lines for early times, before getting a more mixed situation. One can clearly see of how many lines each structure is composed of, and how many nodes there are along each line. For instance, one can compare the times when the transition probability to the last node in each line is significantly enhanced (greater than 0.001, say). This time is comparable to the so-called *hitting time*^118^. For the first graphene network we found that $\pi ^{(1)}_{35,2}>0.001$ for the first time at around $t \approx 15$, while for the second network we have $\pi ^{(2)}_{17,2}(t \approx 6)$, and $\pi ^{(3)}_{11,2}(t \approx 3)$ for the *square* graphene-type network. Thus, generalizing these data we encounter the time needed to reach the last node from the first line to be $\pi _{last,2}(t \approx H_x-2)$. The value for the first graphene is comparable to the value encountered for a discrete circle of *N* nodes^119^, namely $t \approx N/4$. However, in order to see how the graphene geometry influences the spreading, we also determine when we obtain for the first time the value $\pi =10^{-3}$ for the probability to reach the most distant node from 2, which for all three networks is node 70, depicted by a blue square in Fig. 1. For the first network we find $\pi ^{(1)}_{70,2}(t \approx 14.3)$, for the second $\pi ^{(2)}_{70,2}(t \approx 6.8)$, and for the third graphene-type network $\pi ^{(3)}_{70,2}(t \approx 5.4)$. The difference can also be directly related to the number of links of the shortest path between nodes 2 and 70, namely 34 links for the first network, 18 for the second, and 14 for the third. Thus, we also choose to compare the time needed to reach a node from the last line with the same minimum number of links to node 2. We choose that this distance equals 14 links for all three networks. We find the following propagation times: $\pi ^{(1)}_{50,2}(t \approx 4.8)$, $\pi ^{(2)}_{66,2}(t \approx 4.8)$, and $\pi ^{(3)}_{70,2}(t \approx 5.4)$, respectively. From these values we can conclude that the propagation is enhanced when the graphene has longer lines along one direction. Now, we turn our attention to the largest values of the transition probabilities. After comparing all the values for $t \gtrsim 10$ we find that the highest values are: $\pi ^{(1)}_{69,2}(91.3)=0.135$ for the first network, $\pi ^{(2)}_{1,2}(38.0)=0.153$ for the second network, and $\pi ^{(3)}_{12,2}(83.0)=0.209$ for the third network. Higher values of $\pi$ exist for very early times, especially for transitions back to node 2 and its next nearest neighbors. For all three networks we encounter $0.1<\pi <0.2$ for transition probabilities to 6 nodes for the first network, 13 nodes for the second network, and 12 nodes for the third network. Remarkably, usually these nodes belong to the armchain peripheral line that bind node 2 to the first node of the last line, for example for the third network we have nodes 1, 13, 12, 24, 25, 37, 36, 48, 49, 60, and 61. All these findings suggest stronger localization effects for networks $H_x \sim H_y$. At this point it is important to mention that strong localization effects can also be identified when we get a large value for a certain transition probability. For networks with *N* nodes, probabilities which are 10 times higher than the equipartition value 1/*N*, like our networks, can be considered a proof of some localization effects. For some network structures one might find full revivals at certain times^57^ or incomplete revivals^63^. The full revival times $\overline{\tau }$ are determined by solving the equation $\alpha _{j,k}(\overline{\tau })=\alpha _{j,k}(0)$, where $\alpha _{j,k}$ are the transition amplitudes between two nodes given in “Theoretical model”. Full revival times are possible only for very small and particular structures, such as circles and dendrimers, as shown in Refs.^57,119^. Thus, sometimes we focus on computing some incomplete revival times, for which $\pi _{j,j}$ is larger than, say, 0.5. For the honeycomb networks considered in this article we didn’t find such times, having the maximum values far too low to be considered as revival times. For instance, the maximum probability for the first network turns out to be $\pi ^{(1)}_{2,2}(80.3) \approx 0.087$, for the second network is $\pi ^{(2)}_{2,2}(39.3) \approx 0.146$, while for the *square graphene* we found $\pi ^{(3)}_{2,2}(65.8) \approx 0.182$.

By contrast to these findings, in the second row of Fig. 3d–f, we display the time evolution of the probabilities when the CTQW starts from a central node, depicted by a red circle in Fig. 1, namely node 18 for the first network, 27 for the second network, and 30 for the third network. For this choice we encounter similar properties as observed previously for a starting point at the border of the network. First, we compare the times needed by a walker to obtain $\pi =10^{-3}$ for the transition to the most distant node, i.e. node 70. For the first network we find $\pi ^{(1)}_{70,18}(t \approx 7.4)$, for the second graphene structure we have $\pi ^{(2)}_{70,27}(t \approx 2.6)$, and for the third graphene-type network $\pi ^{(3)}_{70,30}(t \approx 2.0)$. These values can be related with the values discussed in panel (a), by remembering that the number of links of the shortest path between the nodes of the three networks is different, namely 18, 9, and 7, respectively. However, for a better qualitative comparison we choose for all networks a node from the last line, which has the same number of links to our chosen central node, let’s say, 7 links. In this case, for the three networks we obtain $\pi ^{(1)}_{59,18}(t \approx 2.0)$, $\pi ^{(2)}_{68,27}(t \approx 1.8)$, and $\pi ^{(3)}_{70,30}(t \approx 2.0)$, respectively. Remarkably, these values show that when the walker starts from the middle nodes the networks with comparable $H_x$ and $H_y$ values, but not equal, show a faster propagation. Now, by comparing all transition probabilities for $t \gtrsim 10$ we encountered the following highest values: $\pi ^{(1)}_{18,18}(91.2)=0.189$, $\pi ^{(2)}_{45,27}(29.0)=0.194$, and $\pi ^{(3)}_{30,30}(26.0)=0.167$, respectively. Thus, only the second network doesn’t have as its maximum probability for a return to the initial node. However, for the second network we see that the second highest probability is $\pi ^{(2)}_{27,27}(43.7)=0.176$, which is exactly the return probability. These values show that for the second network the propagation is faster, but it also shows stronger localization effects. Through a comparative analysis of the results from panels (a) and (b) we can state that faster spreading and stronger localization effects, i.e. higher probabilities for some transitions, are found when the excitation starts from the center rather than from the periphery of the networks. These features are enhanced when the number of hexagons in both directions is comparable.

In the last row of Fig. 3g–i, we show all the transition probabilities $\pi _{j,k}$ for all possible pairs (*j*, *k*) at time $t=10$. Due to the symmetry of the model we have $\pi _{j,k}=\pi _{k,j}$. For the first network, the chosen time is short and the walkers are still localized in the proximity of the starting node; they didn’t have enough time to spread through the whole structure. Thus, one can easily notice a $2 \times 2$ block pattern. The results for the second network (h) show both localization effects, as we clearly see 4 red-colored blocks along the antidiagonal, and good spreading, some red-colored pattern for the rest of the transitions. For the *square* graphene, panel (i), the walkers have enough time to travel through the entire network, thus we encounter a better mixing of the interference waves. These observations are also well supported if one looks at the highest probabilities, which for all three networks are between 0.1 and 0.2. In this interval we encounter 22 pairs of nodes for the first graphene-type network, 4 pairs for the second network, and 6 pairs for the last square graphene sheet network. The maximum value for our three networks also show where the localization effects are a little bit stronger: $\pi ^{(1)}_{35,70}(10)=\pi ^{(1)}_{1,36}(10) \approx 0.144$, $\pi ^{(2)}_{1,1}(10)=\pi ^{(2)}_{54,54}(10) \approx 0.110$, and $\pi ^{(3)}_{6,65}(10) \approx 0.117$, respectively. It is important to mention that these results correspond to a particular value of time, $t=10$, and different findings are possible when other time values are chosen.

**Figure 4:**
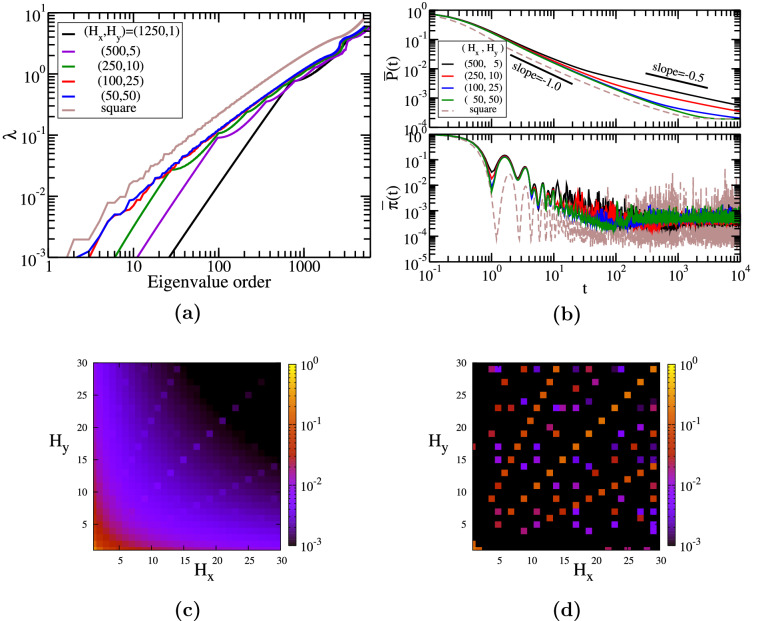
(**a**) Eigenvalue spectrum for rectangular honeycomb networks (graphene sheets), built with parameters $H_x$ and $H_y$, see the procedure described in “Honeycomb networks”. (**b**) Classical and quantum average return probability for these graphene-type networks as a function of time. (**c**) Colour map for the long time average return probability $\chi$ as a function of the parameters $(H_x,H_y)$. (**d**) Relative difference $(\chi -\chi ^*)/\chi$ of the approximate expression $\chi ^*$, again as a function of $(H_x,H_y)$.

In Fig. 4a we display in increasing order the eigenvalues for rectangular honeycomb networks with $(H_x,H_y)=(1250,1)$ (very long, quasi-onedimensional network), (500, 5), (250, 10), (100, 25), and (50, 50) (*square* graphene). The number of nodes for these networks is not the same, ranging between 5002 and 6010, and it depends on the chosen parameters according to Eq. (1). The largest eigenvalue for our first choice equals $\lambda _{N} \approx 5.23$, while for the other networks we encounter 5.90, 5.97, 5.99, and 5.99, respectively. The difference between these largest eigenvalues resides in the specific distribution of degrees and links of the network and their upper bound can be determined from Eq. (14). From this equation we encounter that for our first graphene the upper bound is 5.5, while for the other networks is 6.0. All largest eigenvalues of our networks are greater than the largest eigenvalue of a linear chain, $\lambda _{max} \approx 4$, due to the existence of nodes with a larger number of links. For instance, for a single hexagon we have only three distinct non-vanishing eigenvalues: 1, 1, 3, 3, and 4; while for two coupled hexagons we obtain $\lambda _{10} \approx 4.86$ as the largest eigenvalue. All the networks contain linear segments and nodes with degree 3, thus the eigenvalue spectrum depends on the ratio between them. Graphene-type networks with a very large ratio $H_x/H_y$ show a strong linear chain behavior, following a power-law increase with exponent 2 in the region of low eigenvalues. All the eigenvalues for our choices of parameters $(H_x,H_y)$ are non-degenerate, except the first network, $(H_x,H_y) = (1250,1)$, for which we have one double degenerate eigenvalue, namely $\lambda \approx 3.14$. The most peculiar situation is encountered for *square* graphene networks, for which we get no degeneracy if $H_x$ is even and for odd values of $H_x$ ($H_x \ge 4$) we have always two degenerate eigenvalues: $\lambda =2$ with degeneracy $H_x-2$ and $\lambda =4$ with degeneracy $H_x-3$. These findings will have a big influence on the probabilities not only for graphene, but also for other related honeycomb networks. Some aspects of the eigenvalue spectrum can be understood by recalling the eigenvalues of the square lattice. This parallel is not counterintuitive because our construction procedure resembles a shifted square lattice, if one sees each hexagon as a rectangle. For square network the eigenvalues are given analytically by equation^57,120^:

15 $$\begin{aligned} \lambda _{square}=4-2 \cos \theta _x -2 \cos \theta _y, \end{aligned}$$

with $\theta _x=\frac{2 n \pi }{N_x}$ and $\theta _y=\frac{2 m \pi }{N_y}$, where *n* and *m* are integers and $n \in [0,N_x-1]$ and $m \in [0,N_y-1]$, respectively. Here, $N_x$ and $N_y$ are the number of nodes along $x-$ and $y-$ direction. For *square* networks, i.e. $N_x=N_y$, the angles of both $\cos$ functions are equal, thus we find double degenerate eigenvalues. The eigenvalue $\lambda =4$ is $(N_x-1)-$ fold degenerated and for some particular values of $N_x$, such as $6,12,15,\dots ,$ we find $3-$ and $4-$ fold degenerated eigenvalues. The same $N_x$ values were responsible for the asymmetries observed for the long time average probability $\chi$ for square lattices^120^. Remarkably, also for these networks we encountered discrepancies between even and odd number of nodes along an axis, like our honeycomb graphene networks. For *square* lattices with odd $N_x$ we have $\frac{(N_x-1)^2}{2}$ double degenerate eigenvalues and $N_x$ non-degenerate eigenvalues. For even-numbered $N_x$ we usually have $\frac{(N_x-1)^2+1}{2}$ double degenerate eigenvalues and $N_x-1$ non-degenerate eigenvalues, except the particular values mentioned above. For comparison we plot in Fig. 4a also the eigenvalue spectrum for the square network with $N_x=N_y=71$, which correspond to 5041 nodes and 70 squares along each direction.

In Fig. 4b we show the classical and quantum average probability to return $\overline{P}(t)$, Eq. (10), and $\overline{\pi }(t)$, Eq. (11), as a function of time. Here, we show the results for the last four rectangular shapes of panel (a) and for the square network with 70 squares along each direction. For the classical average probability, the equipartition value, $\overline{P}(t)=1/N$, is easily visible for the *square* graphene sheet. However, also for other networks this value will be reached, but at longer times. This asymptotic time scale is correlated to the length of the linear segments: networks with longer lines, i.e. $(H_x,H_y)=(500,5)$, need longer time to reach the asymptotic behavior. In the intermediate time domain, the average probability decays by following a power-law, $\overline{P}(t) \propto t^{-\gamma }$, with exponent around 1.0, for example $-0.98$ for $(H_x,H_y)=(50,50)$. The duration of this behavior differs, having its maximum exactly for the *square* graphene sheet. Similar behavior was encountered also for single or multilayer dendrimers^57,60^, which are highly symmetric networks composed of nodes with degrees 2 (peripherical) and 3 (inner), like our graphene networks. Differently than dendrimers, the graphene networks have also the circular segments (from the hexagons). The same exponent is also encountered for a square lattice with a similar number of nodes, as shown by a dashed line in the panel. Thus, for networks with longer linear segments the above mentioned behavior is followed by another power-law behavior, $\overline{P}(t) \propto t^{-\zeta }$, with exponent $\zeta$ almost 0.5, like in the case of a single linear chain^57^. For graphene with $(H_x,H_y)=(500,5)$ we determined that the exponent $\zeta$ is 0.46, while for (100, 25) we get 0.31. For our chosen square lattice we do not observe this second power-law behavior. In the region of very short times we encounter an exponential decay $\overline{P}(t) \propto \exp (-\beta t)$, like in the case of dendrimers^60^, but for the graphene networks $\beta$ is higher, $\beta \approx 1.8$. The quantum average return probability shows a less oscillating behavior if we compare with fullerene-type honeycomb networks. For all the networks, due to the influence of linear segments, we observe two distinct behaviors: for times until $t\sim 100$ we see a decay with fluctuations along a power-law with exponent $-1.0$, typical for a ring, a linear chain or square lattice^57^, and for longer times we notice that the fluctuations occur around the long-time average $\chi$. For all graphene networks considered here, we encounter almost the same value: $\chi \approx 0.00019$, which is explained by the fact that for all networks we considered the same number of hexagons, $H_x H_y = 2500$. By following the Euler’s polyhedron formula, see “Honeycomb networks” and Ref.^90^, the difference between the number of links and nodes keeps the same: $Hx Hy - 1$ (where we use Eq. (1) for calculation). The small differences between the curves can be related also to the Link-Node ratio^121^, which is the total number of links divided by the total number of nodes and it gives the connectivity of a network. For our networks this ratio is equal to 1.41, 1.45, 1.47,  and 1.48, respectively. The lowest $\chi$-value, $\chi \approx 0.00016$, corresponds to graphene with $(H_x,H_y)=(500,5)$, which is the network with the longest line-segments. For all four networks, the approximation given by $\chi ^*$ holds, because all the eigenvalues are non-degenerate.

In Fig. 4c we display the long-time average $\chi$, Eq. (13), as a function of the number of hexagons along both axes, $H_x$ and $H_y$. Here, we vary both parameters from 1 to 30, meaning that we consider rectangular graphene sheets of different sizes with the exact number of nodes given by Eq. (1). It is important to stress that the peripheral lines along $H_x$ have a zigzag pattern, while along *y* direction we have an armchair pattern. By comparing two pairs $(H_x,H_y)$ and $(H_x^{'},H_y^{'})$, with $H_x=H_y^{'}$ and $H_y=H_x^{'}$, usually we encounter the same value for $\chi$. However, there are few exceptions for every value of $H_x$, which can be classified into two groups: (i) if $H_x=1$ we get a slightly better efficiency for $(1,H_y)$, i.e. predominantly armchair pattern and (ii) if we fix $H_y=i$ with $i>1$, usually the efficiency is higher for $(H_x,i)$ than for $(i,H_x)$. We remind that better efficiency corresponds to lower values of $\chi$: the maximum efficiency is for $\chi =0$ and a completely inefficient transport for $\chi =1$. It is worth mentioning that the $\chi$-difference between these two networks is very small, it is always less than $10\%$, which is the main reason for having a highly symmetrical map with respect to the diagonal axis, $H_x=H_y$, in Fig. 4c. One can clearly notice that the $\chi$-value gets smaller, which corresponds to better quantum transport efficiency, when we increase the size of the networks, $H_x$ or $H_y$. Remarkably, for some values of the parameter set $(H_x,H_y)$ the efficiency gets lower compared to their adjacent parameter values. This is more visible for some special cases: *(i)* *square* graphene $H_x=H_y$, but only for odd values of $H_x$ ($H_x>7$), (ii) graphene with $(H_x,H_y=2H_x+1)$, and (iii) networks with $(H_x=2H_y+1,H_y)$. Similar behavior was also observed for other 2-dimensional networks: the simple square network, for which it is important to mention whether the number of nodes along an axis is odd or even^120^.

In Fig. 4d we check the validity of the approximation given by $\chi ^*$, by plotting its relative difference to the exact long-time average probability: $\Delta \chi =(\chi -\chi ^*)/\chi$. One can clearly see that the approximation holds very well, $\Delta \chi < 0.001$, for almost all parameter values. However, there are some singular combinations of the parameters for which $\Delta \chi$ is around 0.1, for example networks with $H_x=H_y$ (odd numbers), $(H_x,2H_x+1)$, and $(2H_y+1,H_y)$. The largest values correspond to *square* graphene sheets, with $\Delta \chi$-value getting larger when the network gets larger. For our chosen values the maximum value is encountered for $H_x=29$, namely $\Delta \chi \approx 0.21$, since for even number of hexagons $\Delta \chi =0$.

### Graphite

**Figure 5:**
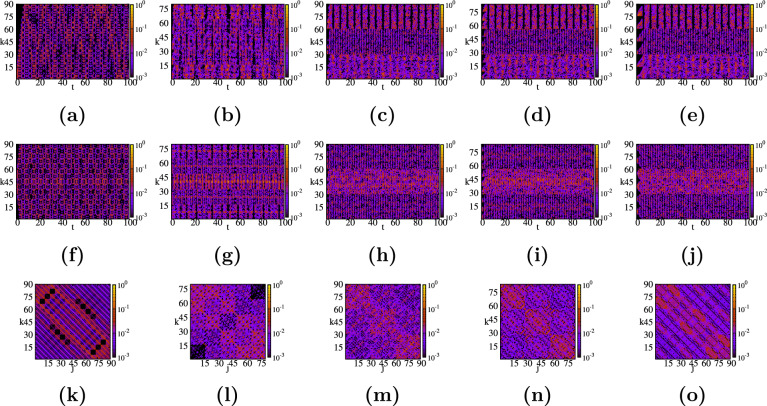
Spacetime structures for five different small graphite-type networks: (**a**,**f**,**k**) long network $(H_x=1, H_y=1, L=15, N=90)$; (**b**,**g**,**l**) medium network $(H_x=2, H_y=2, L=5, N=80)$; (**c**,**h**,**m**) square network $(H_x=3, H_y=3, L=3, N=90)$; (**d**,**i**,**n**) medium intermediate network $(H_x=4, H_y=2, L=3, N=84)$ and (**e**,**j**,**o**) medium thin network $(H_x=7, H_y=1, L=3, N=90)$. For each network we consider $\pi _{k,2}(t)$ (top row), and $\pi _{k,center}(t)$ (middle row) as functions of time, and $\pi _{k,j}(t=10)$ for a fixed time as a function of start and end node (bottom row).

In Fig. 5 we display the contour plots of the quantum average return probability $\pi$, Eq. (7), for the three small graphite-type networks displayed in Fig. 1 and further two graphite-type networks with $L=3$ layers. The number of nodes is variable, ranging between 80 and 90, due to a different number of hexagons along the axes, $H_x$ and $H_y$, and the number of layers, *L*. In order to facilitate the understanding of the results and to compare with simpler graphene sheet networks studied earlier, we continue to use the same numbering method implemented for graphene-type networks, for more details see Fig. 1. Our first network (first column (a), (f), (k)), corresponds to a high network formed by $L=15$ layers, each of them containing a single hexagon, i.e. the three parameters are $(H_x, H_y, L)=(1, 1, 15)$. The second column ((b), (g), and (l)), corresponds to a graphite network with $(H_x, H_y, L)=(8, 3, 5)$. The results for the third graphite, which has the same parameters values $(H_x, H_y, L)=(3, 3, 3)$, are shown in the third column: Figs. 5c,h,m. In order to better understand the influence of the layers, we choose further multilayer honeycomb networks with $L=3$ layers each, namely $(H_x, H_y, L)=(4, 2, 3)$ (fourth column) and (7, 1, 3) (fifth column). To enhance the comparison between networks, we compute the same quantities and we maintain our choice of the threshold value for $\pi$, namely $10^{-3}$, and the time interval, $0<t<100$, with a stepsize of 0.1.

In the first row of Fig. 5, namely (a–e), we show the time evolution of the average quantum transition probability for a walker starting at the peripheral node 2 (blue circle in Fig. 1), for all five networks mentioned above. For the first network, due to the fact that we have 15 layers, i.e. six long lines with 15 nodes each, the linear-like behavior^57^ is obvious. The amplitude waves travel along the network until they bounce off the nodes from the last layer and interfere at longer times. The same process occurs with other chosen networks, but the interference patterns are showing up earlier, because we now have a smaller number of layers, i.e. shorter lines. An interesting situation is encountered for more compact networks, namely our graphite-type networks with only $L=3$ layers. Their patterns for $\pi$ clearly reveal the number of layers and also how many long lines exist in each graphene layer. This is better visible for our last choice, Fig. 5e, for which we clearly notice the $L=3$ layers with 2 long lines each ($H_y= 1$). For these networks we see also the localization effects: each layer showing a distinct pattern, namely a more mixed situation for the starting layer and very low probabilities at some time values for the farthest layer. In order to quantify the differences of quantum transport in these networks, we determine when the transition probability to a node with the same distance (same number of links) exceeds for the first time a threshold value, thus experiencing the most of the network topology. For this, we choose as threshold the value $\pi =0.001$ and the chosen distance is 10 links. Having different parameters forces us to choose different node numbers for each of the five networks, obeying the condition to be 10 links away from node 2. For the first three networks we depict these nodes by blue squares in Fig. 1, while for the last two networks we consider node 84. For all five graphite-type networks we found that the transition probability is higher than 0.001 for the first time at the following times: $\pi ^{(1)}_{47,2}(t \approx 2.4)$, $\pi ^{(2)}_{80,2}(t \approx 2.1)$, $\pi ^{(3)}_{90,2}(t \approx 3.1)$, $\pi ^{(4)}_{84,2}(t \approx 3.2)$, and $\pi ^{(5)}_{84,2}(t \approx 2.5)$, respectively. Thus, we can conclude that networks with longer lines allow a faster spreading. These lines can be part of a single layer, like structure 5, or they can link various layers, like the first network. Regarding the highest values of the transition probabilities for each network, we found out that the highest values are: $\pi ^{(1)}_{2,2}(31.4)=0.383$, $\pi ^{(2)}_{59,2}(38.0)=0.435$, $\pi ^{(3)}_{25,2}(69.4)=0.280$, $\pi ^{(4)}_{2,2}(69.0)=0.259$, and $\pi ^{(5)}_{2,2}(31.4)=0.290$, respectively. All these values show that networks with faster spreading show also stronger localization effects, which correspond to higher values of $\pi$. The second network shows the strongest localization effects, which is a consequence of the fact that it has the highest Link-Node ratio^121^: 1.98, which suggest a higher connectivity. This high value is mainly due to links between layers, since its Link-Node ratio of a single layer is the second lowest from the five networks. Thus, the fast propagation along the network is due to spreading on one layer, while the high number of links between layers is responsible for localization. The most compact network, $(H_x=3, H_y=3, L=3)$, shows slow spreading and medium localization effects. For very short times one encounters higher $\pi$-values for all the networks, but we skipped these values for aiming to a situation in which the waves have bounced off the last layer. For all these networks we consider probabilities $\pi >0.1$ and we found such values for transitions to 7 nodes (first network), 38 nodes (second network), 2 nodes (third network), and 1 node for each of the last two graphite-type networks. Remarkably, networks with more layers show also higher values of the transition probabilities, but also more nodes have values above 0.1 at some time value. Thus, we can state that a faster spreading and stronger localization effects are enhanced by the addition of layers, which can be easily verified by comparing to the results of graphene. For our small graphite networks we didn’t find any total or partial revival times. The maximum probabilities for the five networks are $\pi ^{(1)}_{2,2}(31.4) \approx 0.383$, $\pi ^{(2)}_{2,2}(82.0) \approx 0.285$, $\pi ^{(3)}_{2,2}(24.8) \approx 0.225$, $\pi ^{(4)}_{2,2}(69.0)=0.259$, and $\pi ^{(5)}_{2,2}(31.4)=0.290$, respectively.

In the second row of Fig. 5, namely (f–j), we display the transition probabilities from a central node, $\pi _{k,center}(t)$, for all five small graphite-type networks. Here, we choose transitions from one of the nodes situated in the middle of each network, more precisely, one node from the center of the middle layer. Each choice is depicted by red color in Fig. 1, i.e. the node numbers are: 43, 40, 41, 43, and 38, respectively. Similar properties as for transitions from the first layer are observed. For long networks, like the first two networks, we notice similar patterns as a wave propagation on a line, while for more compact networks one can easily see some localization properties at nodes from the middle layer. The localization effects are stronger if the walker starts from the middle layer, which can also be seen by comparing the highest transition probability to return at the starting node. For instance, the highest values of the probabilities for each network are: $\pi ^{(1)}_{48,43}(39.9)=0.413$, $\pi ^{(2)}_{41,40}(22.3)=0.477$, $\pi ^{(3)}_{41,41}(23.3)=0.346$, $\pi ^{(4)}_{42,43}(58.8)=0.404$, and $\pi ^{(5)}_{38,38}(39.9)=0.508$, respectively. All these values are larger than the maximum probabilities found for walkers starting at the peripheral node 2, having the relative increase ranging from $7.8\%$ to $75\%$. The largest increase corresponds to the last network, which is peculiar since its overall connectivity measure, the Link-Node ratio, equals 1.86, being the lowest from all five networks. Thus, for this particular network the starting point of the walker has a crucial importance. The same is valid if one compares the maximum value of the probability to return to the starting node, for which we get $\pi ^{(1)}_{43,43}(31.4) \approx 0.386$, $\pi ^{(2)}_{40,40}(50.7)=0.464$, $\pi ^{(3)}_{41,41}(23.3)=0.346$, $\pi ^{(4)}_{43,43}(35.6)=0.343$, and $\pi ^{(5)}_{38,38}(39.9)=0.508$, respectively. Now, we consider probabilities $\pi >0.3$ and for each network we encountered two such transitions, namely back to the starting node and to one of their nearest neighbors. This represents a big increase, more than double if compared to similar small graphene networks, and it shows that by increasing the number of layers we increase the velocity of the propagation, but also the localization effects become more pronounced.

In the last row of Fig. 5k–o, we show all possible transition probabilities $\pi _{j,k}$ for a fixed time $t=10$. For the first graphite-type network. the walkers that started from the first layer have high probabilities to be at one node from the last 4 layers. The walkers starting at the second layer have high probability to be at a node from the last 3 layers and they have $\pi <0.001$ to be localized at layer 12. We have faster spreading along the long lines that connect the layers and the complete mixed situation was not reached, due to a still short propagation time, $t=10$. For the second network the localization effects, which can be related to higher values of $\pi$, thus, more red coloured points, are more present, making more visible the $5 \times 5$ block pattern. Also in this case at $t=10$, the walkers that started from the first layer have higher probabilities to be at the last layer. For the three-layered graphite networks the walkers visited all the nodes, thus one can clearly notice a better mixing of the waves. By comparing the last three networks, we notice a better spreading when the rectangular graphene sheet structure is not that *square*. We have probabilities higher than 0.1 only for 18 pairs of nodes (network with $(H_x=3, H_y=3, L=3)$), 4 pairs (network with $(H_x=2, H_y=3, L=4)$), and 0 for the network with $(H_x=7, H_y=1, L=3)$. The maximum values for these three networks also give a hint where the localization is stronger: $\pi ^{(3)}_{1,76}(10) \approx 0.138$, $\pi ^{(4)}_{3,82}(10) \approx 0.109$, and $\pi ^{(5)}_{2,71}(10) \approx 0.086$, respectively. Thus, we can conclude that a graphite-type network with $H_x=H_y$ shows a slower spreading than other networks, but the addition of layers increases the spreading.

**Figure 6:**
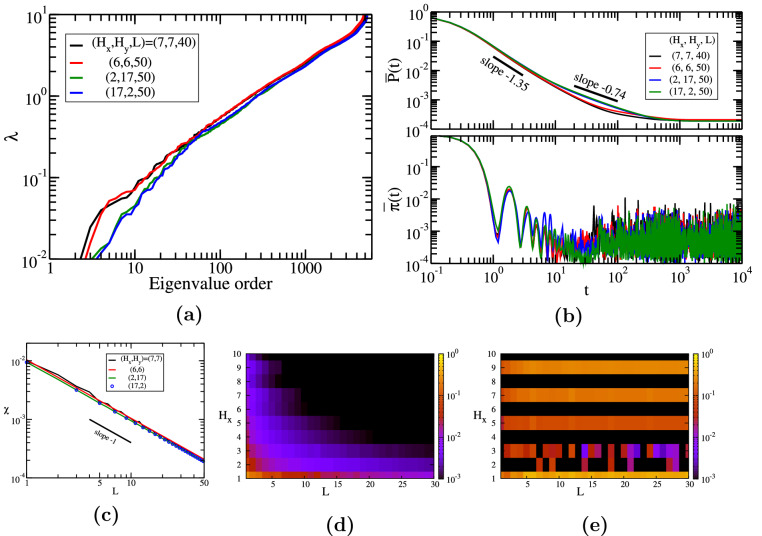
(**a**) Eigenvalue spectrum for graphite-type networks. (**b**) Classical and quantum average return probability. (**c**) Long time average probability $\chi$ as a function of the number of layers *L*. (**d**) $\chi$ as a function of $(L,H_x)$ for square graphene sheet layers ($H_x=H_y$). (**e**) Relative difference $(\chi -\chi ^*)/\chi$ as a function of $(L,H_x)$ for square graphene layers.

In Fig. 6a we display in increasing order the eigenvalues of four graphite-type networks: two of them have a *square* graphene sheet as base, namely $(H_x, H_y, L, N)=(7, 7, 40, 5040)$ and (6, 6, 50, 4800), one has a predominantly armchair pattern $(H_x, H_y, L, N)=(2, 17, 50, 5300)$ and the last one has a predominantly zigzag pattern $(H_x, H_y, L, N)=(17, 2, 50, 5300)$. The largest eigenvalues for our networks are: $\lambda ^{(1)}_{N} \approx 9.90$ and $\lambda ^{(2)}_{N} \approx 9.87$ for the first two networks, $\lambda ^{(3)}_{N} \approx 9.71$ for the *armchair* graphite, and $\lambda ^{(4)}_{N} \approx 9.63$ for the *zigzag* network. Similar to graphene, the difference between the highest eigenvalues is due to the degree distribution and the particular distribution of links of each network. The upper bound of the largest eigenvalue, as calculated from Eq. (14), is equal to 10.0 for all these multilayer networks. The eigenvalue spectrum of the multilayer network formed by *square* graphene with $H_x=H_y=7$ is degenerate: two double degenerate eigenvalues, 39 eigenvalues with degeneracy 4, 36 eigenvalues with degeneracy 5, 3 eigenvalues with degeneracy 6, and one eigenvalue with degeneracy 11. The other considered graphite structure composed of *square* graphene has only non-degenerate eigenvalues. The last two considered networks have only a few double degenerate eigenvalues, namely $\lambda =2$ and 4 for $(H_x, H_y, L)=(2, 17, 50)$ and the eigenvalue $\lambda =2$ for $(H_x, H_y, L)=(17, 2, 50)$. Before explaining these differences we stress that the whole spectrum of the eigenvalues of a graphite-type network can be determined by knowing the eigenvalues of a single graphene. Let’s define the eigenvalue spectrum of the graphite as $\Lambda =(\Lambda _0, \Lambda _1, \dots , \Lambda _{L-1})$, where *L* is the number of layers and each $\Lambda _j$ contains $N_L$ eigenvalues, where $N_L$ is the number of nodes from a single layer. The eigenvalues of each $\Lambda _j$ can be written as^60,122^:

16 $$\begin{aligned} \Lambda _j= 2-2 \cos \left( \frac{j \pi }{L} \right) + \lambda _i^{'}, \end{aligned}$$

where $\lambda _i^{'}$ are the eigenvalues of a graphene sheet, with $i=1,\dots ,N_L$. From the last equation we find that the eigenvalues of a very long graphite range between $\lambda _i^{'}$ and $\lambda _i^{'}+4$. This gives another explanation for the largest eigenvalues being limited to 10. For *square* graphene sheet, see the discussion of Fig. 4a, we notice that if $H_x$ is even there is no degeneracy, while if $H_x$ is odd we have two eigenvalues with degeneracy $H_x-2$ and $H_x-3$, respectively. The addition of layers will increase the number of degenerated eigenvalues (if exists) or increase the degeneracy of some existing eigenvalues, as can be inferred from Eq. (16).

In Fig. 6b we show the results for the classical and quantum average probability to return to the starting node $\overline{P}(t)$, Eq. (10), and $\overline{\pi }(t)$, Eq. (11). We consider the same networks studied in panel (a). For all the networks, the classical average probability of equipartition, $\overline{P}(t)=1/N$, is reached at almost the same time $t \approx 500$. In the intermediate time domain, $\overline{P}(t)$ follows a power law with an exponent around $-1.35$ for all the networks. For the last two networks, we encounter a second power-law behavior with exponent equal to $-0.74$. Similar behavior was obtained also for modified multilayer spiderwebs^63^, more exact a power-law decay with the exponent $-1.3$, which is followed by another power-law decay with exponent $-0.38$ for a sufficiently large number of layers. The difference in the exponents resides in the fact that the degrees of the nodes for the modified spiderwebs range between 2 and 7, while for graphite we have degrees between 3 and 5. The quantum average return probability shows similar behavior for all the networks, but with stronger oscillations than for a single layer of graphene. This is due to an increase in the length of linear segments for a graphite structure with 40 or 50 layers. For times longer than $t \gtrsim 10$ we observe the usual fluctuations around the long-time average $\chi$, which is ranged between 0.00018 (last two networks) to 0.00025 (first network). These values resemble the $\chi$-values of a graphene with a similar number of nodes, see the text corresponding to Fig. 4a for more details. For the network composed of *square* graphene with an odd number of hexagons, the approximate value $\chi ^*$ fails; we determine that the relative difference turns out to be $(\chi -\chi ^*)/\chi \approx 0.19$. For the other networks the approximation holds much better, having a relative differences lower than $10^{-3}$. This result can be related to the degeneracy of the eigenvalues, which was discussed in the previous panel.

In Fig. 6c we monitor how $\chi$ is influenced by a progressive increase in the number of layers. In order to generalize the results, we consider the four basic graphene sheets of the previous panels, but we vary the number of layers from 1 to 50. Remarkably, for all networks, we get almost the same behavior, confirming our previous findings that the quantum transport for graphene based multilayer networks is mainly dependent on the number of nodes rather than on the number of hexagons along the *x* or *y* axes. All the curves follow a power-law decay with exponent $-1$, i.e. $\chi \propto L^{-1}$, meaning that one of the mechanism to increase the quantum efficiency is stacking graphene layers on top of each other. The same behavior was also found for other similar networks, such as multilayer dendrimer networks^60^, for which the power-law exponent equals $-0.8$. Thus, the graphite networks show a faster improvement of the quantum efficiency, which is due to the intralayer lines: the honeycomb lattices.

In Fig. 6d we focus on the long-time average $\chi$, Eq. (13), but we consider only the graphite-type networks having as basic graphene sheet a *square* $H_x=H_y$. We show the results for networks with $H_x=H_y=1$ to 10 and the number of layers ranges from 1 to 30. Thus, we have graphite-type networks of very different node numbers, from 6 to 7200, values verified by using Eqs. (1) and (2). For all the values of $H_x$ one can clearly notice that the $\chi$-value gets lower when we increase the number of layers, on average we get a decrease around 30 if we go from a single layer structure to a 30-layer graphite, which also confirm the results from the previous panel. By increasing the number of hexagons on both axes from $H_x=1$ to 10 on average we increase the quantum efficiency, measured through $\chi$, by more than 70 times. This is due to the fact that the number of nodes increases linearly with the number of layers and more than quadratically with the number of hexagons along an axis. The same $\chi$-value can be found for distinct parameter values, as can be easily visualized by following the same color tone in the figure. Thus, it is possible to encounter the same efficiency by varying both $H_x$ and *L* at the same time, depending on the most suitable option.

In Fig. 6e we monitor the relative difference $\Delta \chi$ for the same parameter values as the previous panel. We notice two distinct behaviors: the approximation is valid, $\Delta \chi <0.001$, for odd $H_x$-value and the approximation doesn’t hold for even $H_x$. This peculiar result is found for all $H_x \ge 4$ independent of the number of layers and it is a direct consequence of the eigenvalue spectrum of a graphene sheet. As discussed in Figs. 4a and 6a we have degenerate eigenvalues only for odd-numbered $H_x$. The highest value for our choice of parameters is $\Delta \chi \approx 0.265$, corresponding to a *square* graphene-type network with $H_x=9, L=30$, but larger values can be found when the number of layers is increased or the number of hexagons along each axes. Remarkably, for $H_x=H_y=2$ or 3 we have a strong dependence on the number of layers and both situations, namely a valid or invalid approximation, can be found.

### Nanotube networks

**Figure 7:**
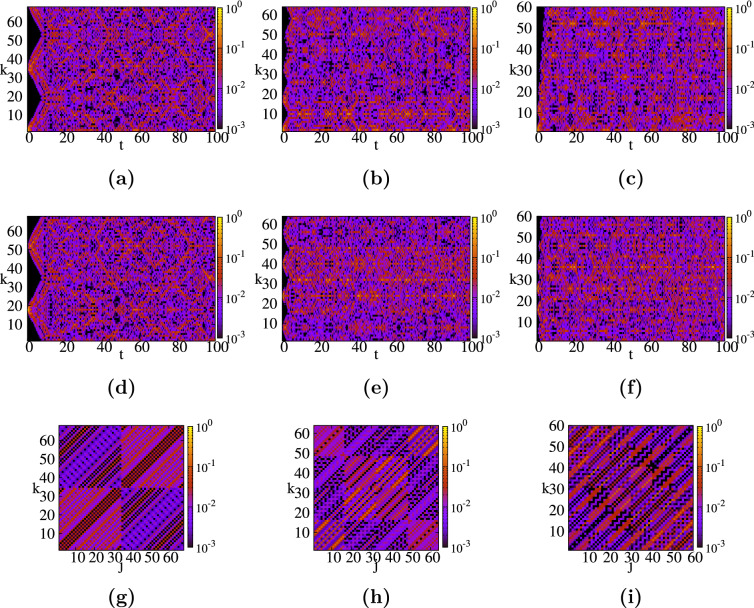
Spacetime structures for small nanotube-type honeycomb networks corresponding to three types of graphene structures considered in Fig. 3: (**a**,**d**,**g**) long networks $(L_x=34,L_y=2,N=68)$; (**b**,**e**,**h**) medium networks $(L_x=16,L_y=4,N=64)$; (**c**,**f**,**i**) large networks $(L_x=10,L_y=6,N=60)$. For each network we consider the quantum transition probabilities $\pi _{k,1}(t)$ (top row), $\pi _{k,middle}(t)$ (middle row), $\pi _{k,j}(t=10)$ (bottom row).

In Fig. 7 we show the contour plots of the quantum average return probability $\pi$, Eq. (7), for three small nanotube-type networks: $(L_x,L_y,N)=(34,2,68)$, (16, 4, 64), and (10, 6, 60). Here, the parameters $L_x$ and $L_y$ correspond to the number of nodes along the *x*- and *y*-axis, respectively. In order to easier understand the results it is important to remember that $\frac{L_x}{2}=H_x$ and $L_y-1=H_y$. For an easier understanding of the influence of rolling up the graphene sheets we choose the same networks as in Figs. 1d–f and 3. The first nanotube structure corresponds to a large and thin network: 17 hexagons along the *x*-axis and we present its results in the first column. In the second column, panels (b), (e), and (h), we display the results for a nanotube structure with $(L_x, L_y, N)=(16, 4, 64)$. The last nanotube-type network is formed from a *square* graphene sheet with $(L_x, L_y, N)=(10, 6, 60)$, which is also depicted in Fig. 1 and its results are shown in the third column, Fig. 7c,f,i.

In the first row of Fig. 7, namely (a–c), we display the time evolution of the quantum transition probability of a walker starting at the peripheral node 2, $\pi _{k,2}(t)$. The first nanotube structure is basically formed by two long rings of 34 nodes, coupled to each other by 18 links, thus we observe a similar behavior as for the long thin graphene-type network. The difference is that the amplitude waves do not bounce off, but they continue to travel along the ring and for times longer than 10 we get stronger interference. The same behavior is encountered for the last two considered nanotube structures, but for these networks the localization effects are even more visible. Now, we consider the minimum time needed to reach a value $\pi =0.001$ for walks with the same distance (same number of links) along the network, namely 10 links. For this, we choose a node from the other peripheral line, which for the third network is 57, depicted by a blue square in Fig. 1. For the three nanotube networks we found the following times: $\pi ^{(1)}_{47,2}(t \approx 4.5)$, $\pi ^{(2)}_{57,2}(t \approx 3.5)$, and $\pi ^{(3)}_{57,2}(t \approx 3.7)$, respectively. Remarkably, the nanotube structures with longer rings are not favored to reach a certain probability value first, but networks with more similar values for $L_x$ and $L_y$. This result is different than the graphene networks and it is a direct consequence of rolling up the graphene sheet, transforming the lines into circles. For the three nanotube structures the highest transition probabilities in the region $10<t<100$ are $\pi ^{(1)}_{53,2}(97.0)=0.223$, $\pi ^{(2)}_{2,2}(40.8)=0.346$, and $\pi ^{(3)}_{52,2}(93.6)=0.337$, respectively. All these values are higher than the values for similar graphene networks, suggesting that the localization effects are stronger, despite the circular lines. Now, we consider only the values $\pi >0.2$ and we encounter them only for transitions to node 53 (first network), to nodes 2 and 10 (second network), and to nodes 2, 47, and 52 (third network). The net values for the last two networks are higher, confirming a more pronounced localization pattern. For our small nanotube-type networks we did not find any total or partial revival, the maximum probability being equal to $\pi ^{(1)}_{2,2}(56.7) \approx 0.180$, $\pi ^{(2)}_{2,2}(91.3) \approx 0.330$, and $\pi ^{(3)}_{2,2}(79.7) \approx 0.210$, respectively.

In the second row of Fig. 7, namely (d–f), for each network we show the transition probabilities from one of the most central nodes, $\pi _{k,center}(t)$. For example, we choose node 26 for the third nanotube structure and we depicted it by red color in Fig. 1. We observe similar behavior as in panels (a)-(c), namely faster propagation and stronger localization effects for the last two nanotube networks. For instance, the largest values of the probabilities for each network are: $\pi ^{(1)}_{35,18}(97.0)=0.232$, $\pi ^{(2)}_{24,24}(91.3)=0.343$, and $\pi ^{(3)}_{36,26}(93.7)=0.346$, respectively. All these values are comparable to the maximum probabilities found for walkers starting at a peripheral node. The same is valid if one compares the maximum probabilities to return to the origin: $\pi ^{(1)}_{18,18}(56.7) \approx 0.180$, $\pi ^{(2)}_{24,24}(91.3)=0.343$, and $\pi ^{(3)}_{26,26}(95.0)=0.232$, respectively. Thus, by comparing all these values, we can state that for nanotube networks the starting point of a walker is largely irrelevant, which is different than the graphene networks.

In the last row of Fig. 7g–i, we show the transition probabilities $\pi _{j,k}$ at a fixed time, $t=10$. For all three nanotubes one can see how many lines there are along the *y*-axis, due to the block structure arrangement. For the first two nanotube networks, walkers that start from one line have higher transition probabilities to a node from the same line. For the last nanotube structure, the walkers have the highest probabilities to be at a different line, for example, nodes from the first line are coupled to nodes from the last line, nodes from the second line are coupled to nodes from the fourth line and the nodes from the third line are paired with nodes from the fifth line. We find probabilities larger than 0.1 only for the last two nanotube networks, namely 32 pairs of nodes for the second and 60 pairs for the third nanotube structure. The largest values for these networks also show us where we have stronger localization effects: $\pi ^{(1)}_{i,i+17}(10)=\pi ^{(1)}_{i+17,i}(10) \approx 0.062$ with $1 \le i \le 17$ and $35 \le i \le 51$, $\pi ^{(2)}_{i,i+8}(10)=\pi ^{(2)}_{i+8,i}(10) \approx 0.158$ with $1 \le i \le 8$ and $49 \le i \le 56$, and $\pi ^{(3)}_{i,j}(10) \approx 0.142$ for 10 pairs of nodes, respectively. Thus, a nanotube-type network derived from a *square* graphene with $L_x/2=L_y-1$ doesn’t show any more the slowest spreading and the strongest localization effects, due to the rolling up mechanism.

**Figure 8:**
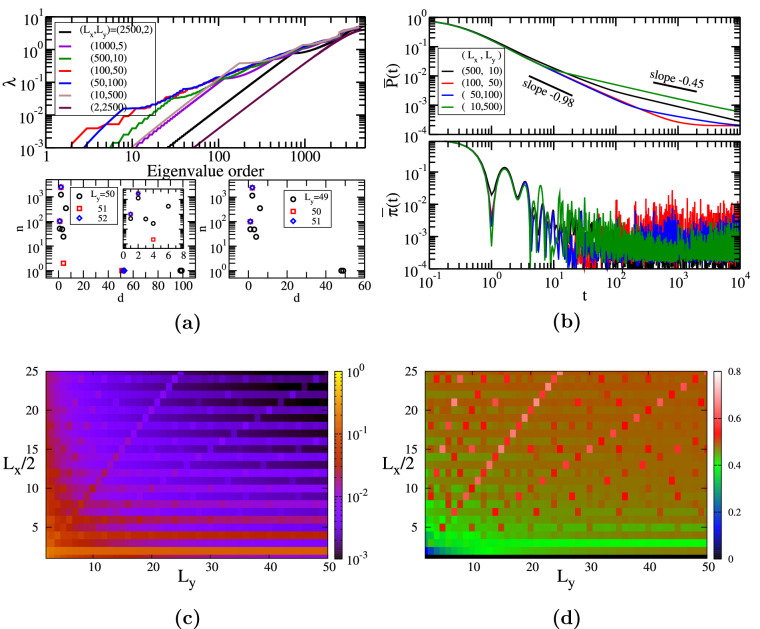
(**a**) Eigenvalue spectrum for nanotube networks with $N=5000$ (top). Eigenvalue degeneracy *n*(*d*) for nanotube structures with $L_x=100$ (bottom left) and $L_x=98$ (bottom right). (**b**) Classical and quantum average return probability for nanotubes with $N=5000$. (**c**) Long time average probability $\chi$ as a function of $(L_x/2,L_y)$. (**d**) Relative difference $(\chi -\chi ^*)/\chi$ as a function of $(L_x/2,L_y)$.

In Fig. 8a we plot in double logarithmic scale the eigenvalue spectrum of nanotube-type networks with $N=5000$ and various values of $(L_x,L_y)$, see “Honeycomb networks” for more details. Here, we display for comparison the eigenvalues of a single line, $(L_x,L_y)=(2,2500)$. For this particular case we get a non-degenerate spectrum, with eigenvalues ranging from 0 to 4. For $(L_x,L_y)=(2500,2)$, i.e. basically two rings coupled to each other through 1251 links, we encounter mainly 2-fold degenerate eigenvalues, more precisely 2497 distinct eigenvalues and a 3-fold degenerate eigenvalue, $\lambda =3$. This feature is related to the rings present in the nanotube structure and it is more evident when $L_y$ increases. For the other nanotube networks we always have the eigenvalues 2 and 4 with a higher degeneracy, as it was found for graphene and graphite. Their highest degeneracy is encountered for the nanotube with $(L_x,L_y)=(100,50)$, for which we obtain the degeneracy 98 for $\lambda =4$ and 99 for $\lambda =2$. It is important to remember that the most *square* network corresponds to $L_x/2=L_y-1$, for example $(L_x,L_y)=(100,51)$. Additionally, the highest eigenvalue increases to 5.23 for $(L_x,L_y)=(2500,2)$, 5.86 for (1000, 5), 5.96 for (500, 10), and to almost 6 for other considered values. This value is related to the particular degree and link distribution of each network and the estimation for the upper bound of the largest eigenvalue, Eq. 14, gives 5.5 for (2500, 2) and 6.0 for other networks. In the bottom of Fig. 8a we plot the eigenvalue degeneracy for nanotube structures with $L_x=100$ (left) and $L_x=98$ (right). We focus on *square* networks, $L_x/2=L_y-1$, and we consider networks having the same number of nodes along the *x*-axis, $L_x$, and a variable number of nodes along the *y*-axis. We observe that networks with $L_x/2=L_y$ have the most discrete spectrum, while for the other nanotubes we encounter two situations: (i) for *odd* $L_x/2$ we have only single or doubly degenerate eigenvalues and (ii) for *even* $L_x/2$ there are single, double, and sometimes 4-fold degenerate eigenvalues, while $\lambda =2$ is ($L_y+1$)-fold degenerate and $\lambda =4$ is $L_y$-fold degenerate. For nanotube networks with $L_x/2=L_y$ ($L_y>6$) we always have eigenvalues of degeneracy 1, 2, 3, 4, and 6 and the highest degenerate eigenvalues are $\lambda =2$ and $\lambda =4$. The degeneracies of these two eigenvalues are equal to $L_y-1$ and $L_y$ for networks with *odd* $L_y$ and $2L_y-1$ and $2L_y-2$ for networks with *even* $L_y$. Similar differences were spotted also for *square* graphene and they are due to the symmetry of the networks. For instance, nanotubes with even-numbered $L_y$ (or graphene with odd $H_y$) have as center of the network a hexagon, for example the hexagon formed by nodes 29, 30, 31, 41, 42,  and 43 for the graphene depicted in Fig. 1e. Thus, the asymmetry is increased since any of the above mentioned nodes can be considered as the center of the network. For nanotubes with odd-numbered $L_y$ (or graphene with even $H_y$) we have only 2 nodes that can be considered in the center of the network.

In Fig. 8b we plot the classical and quantum average return probability $\overline{P}(t)$, Eq. (10), and $\overline{\pi }(t)$, Eq. (11). We consider networks with $(L_x, L_y)=(500, 10)$ and (100, 50) and the same numbers, but interchanged. For the classical probability all the networks will reach the equipartition value, $\overline{P}(t)=1/N$, but at different times. Longer networks, i.e. $L_x=500$ and $L_y=500$, need more time to reach this value following a power-law decay with exponent 0.45, which is close to the value encountered for linear chains, 0.5. For intermediate time values we encounter another power-law decay of exponent 0.98, which is similar with the exponent encountered for graphene or dendrimers, and it is influenced mainly by the nodes with degree 3. The quantum average return probability displays stronger oscillations than those for a graphene-type network for all nanotube networks, which is due to an increase in size of linear segments. For times longer than 30 we observe fluctuations around the long-time average $\chi$. From top to bottom the $\chi$-value is equal to 0.0004, 0.0015, 0.0005,  and 0.0003, respectively. Thus, the nanotube structure with $L_x/2=L_y$ is less efficient than the other networks, as can be inferred also from the eigenvalue spectrum. Remarkably, for all these nanotube structures the approximation, $\chi ^*$, doesn’t hold; we encounter values of the relative difference $(\chi -\chi ^*)/\chi$ ranging from 0.445 to 0.608.

In Fig. 8c we display the long-time average $\chi$, Eq. (13), as a function of the number of nodes along both axes, more precisely $L_x/2$ and $L_y$. Here, we vary both parameters from 1 to 50, meaning that we have nanotube networks of different sizes, with the number of nodes given by $N=L_x L_y$. Due to our construction we have only zigzag nanotube structures on both peripheral lines. We observe two distinct behaviors when $L_y$ is fixed and $L_x/2$ is varied, namely a lower value for $\chi$ if $L_x/2$ is odd when compared to even $L_x/2$. When we increase $L_y$, the $\chi$-value gets lower for nanotube networks with the same $L_x/2$, but there are some exceptions: (i) nanotubes with $L_x/2=L_y$, being more evident for even numbers of $L_x/2$, (ii) nanotubes with $L_x/2=2L_y$, and (iii) nanotubes with $L_x=L_y$ for even number of $L_x/2$. Remarkably, for every value of $L_x/2$ there are other additional values of $L_y$ for which the value of $\chi$ does not maintain its descending trend. These exact values depend on $L_x/2$, but usually it happens when $L_x/2$ or $L_y$ is a multiple of the other or when they share a greatest common divisor larger than 4, being related with the internal symmetry of the nanotubes.

In Fig. 8d we monitor the relative difference $\Delta \chi$ for the same nanotube structures as in the previous panel. Remarkably, for all the networks the approximation is not valid, $\Delta \chi >0.12$, except the particular case $L_x/2=1$, which corresponds to a single line. For our considered values of $L_x$ and $L_y$, we encounter that the largest value is $\Delta \chi \approx 0.68$, corresponding to the nanotube network with $L_x/2=L_y=21$. For almost all $L_x/2$ we find that the largest value of $\Delta \chi$ corresponds to networks with $L_x/2=L_y$. Large values are encountered also for the same nanotube networks mentioned in the discussion of the previous panel.

## Discussion

In this work we have studied continuous-time quantum walks on honeycomb networks. We consider fullerene-type, graphene-type, graphite-type, and nanotube-type networks. We have investigated the average return probability and the long-time average transition probability $\chi$, which in our model can be reduced to the complete determination of the eigenvalues and/or the eigenvectors of the connectivity matrix. For all honeycomb networks we observed a non-trivial interplay between strong localization effects and good spreading. The spreading can be enhanced if we increase the linear segments, which can be done through two mechanisms. The first one is given by stacking identical graphene sheets, thus, creating a graphite network, for which we are able to obtain an increase of quantum transport efficiency proportional to the number of layers. The second mechanism corresponds to an increase of the number of hexagons only along one direction, which was shown to hold for graphene, graphite or nanotube structures. For fullerenes we found that $C_{70}$ allows for better quantum transport, as its long-time average return probability is three times lower than for a $C_{60}$-structure. The explanation resides in a symmetry breaking of the transition from $C_{60}$ to $C_{70}$. Due to a higher symmetry $C_{60}$ shows higher probabilities also for transitions to diametrically opposite nodes, differently than $C_{70}$. For all considered honeycomb networks, we observe little differences, less than $10\%$, between networks with predominant armchair and zigzag pattern. We have encountered the same quantum efficiency for different values of the parameters of a certain type of network, which allows us to choose a most convenient setup. A peculiar situation is given by networks with the same number of hexagons along both directions, which we called *square* networks. In this case, there are differences between an even or odd number of hexagons, a situation reminiscent of the square lattices into which we can map our graphene networks. We have shown that for *square* honeycomb networks with an odd number of hexagons along one direction the quantum efficiency is diminished when compared to adjacent parameter values. Due to the rolling up mechanism the decrease in efficiency occurs for nanotube composed of almost *square* graphene networks. A similar decrease in efficiency, but not so evident, has been encountered also for graphene or nanotube structures with the number of hexagons along one direction being a multiple of the hexagons along the other direction. For graphite this behavior is accentuated by the increase in the degeneracy of some eigenvalues, which is a direct consequence of stacking layers on top of each other. For graphene and graphite, but different from nanotubes, we have found that when placing the initial excitation in the center of the networks we get faster spreading, but also stronger localization effects. Thus, the transport along such networks depends on the initial condition. However, this dependence is not too strong and it can be overcome when the walkers start from a superposition of states, making these structures feasible also for applications, such as quantum search algorithms or quantum information. Strong localization properties were observed for discrete-time Grover-type quantum walks on honeycomb networks^81^. In this model, the localization disappears if the initial coin state is a superposition of all eigenstates. Using the same model, it was shown in Ref.^86^ that longer and thinner nanotubes exhibit better transport, which we also observed in our model and in the staggered quantum walk on the infinite hexagonal lattice^85^. For graphene based networks we observed a mixed situation regarding the validity of the approximation $\chi ^*$. It is not valid for fullerene- and nanotube-type networks, but it is valid for the majority of graphene networks, except for some special parameter values, for instance the *square* graphene with an odd number of hexagons along one direction. We have shown that for graphite formed from *square* graphene sheets ($H_x>3$) the approximation holds for an even number of layers, but it fails for an odd number of layers.

We expect our results to be of interest in various research areas, such as quantum transfer of excitons, quantum transport on graphene and related structures, quantum information or quantum search algorithms. Our findings can be exploited in experiments involving the walk of a single particle on tailored graphene-like networks. Experimental quantum walks of individual or multiple particles have been realized for various platforms, including photons^33,37,38,43,123–125^, nuclear magnetic resonance^126^, trapped ions^34–36,127,128^, neutral atoms^129,130^, or superconducting qubits^131,132^.
